# Combined Kinetic Studies and Computational Analysis on Kojic Acid Analogs as Tyrosinase Inhibitors

**DOI:** 10.3390/molecules19079591

**Published:** 2014-07-07

**Authors:** Carlyle Ribeiro Lima, José Rogério A. Silva, Érica de Tássia Carvalho Cardoso, Edilene O. Silva, Jerônimo Lameira, José Luiz Martins do Nascimento, Davi do Socorro Barros Brasil, Cláudio N. Alves

**Affiliations:** 1 Laboratório de Planejamento e Desenvolvimento de Fármacos, Instituto de Ciências Exatas e Naturais, Universidade Federal do Pará, 66075-110 Belém, PA, Brazil; E-Mails: carlylelima@hotmail.com (C.R.L.); rogerio@ufpa.br (J.R.A.S.); lameira@ufpa.br (J.L.); davibb@ufpa.br (D.S.B.B.); 2 Programa de Pós-Graduação em Biotecnologia, Universidade Federal do Pará, 66075-110 Belém, PA, Brazil; 3 Laboratório de Neuroquímica Molecular e Celular, Instituto de Ciências Biológicas, Universidade Federal do Pará, 66075-110 Belém, PA, Brazil; E-Mail: tassia.10@gmail.com; 4 Laboratório de Biologia Estrutural, Instituto de Ciências Biológicas, Universidade Federal do Pará, 66075-110 Belém, PA, Brazil; E-Mail: edilene@ufpa.br; 5 Instituto de Tecnologia, Universidade Federal do Pará, 66075-110 Belém, PA, Brazil

**Keywords:** tyrosinase, kojic acid, kinetic assays, inhibition, molecular docking, molecular dynamics, binding free energy, LIE

## Abstract

Tyrosinase is a key enzyme in melanin synthesis and widely distributed in plants and animals tissues. In mammals, this enzyme is related to pigment production, involved in wound healing, primary immune response and it can also contribute to catecholamines synthesis in the brain. Consequently, tyrosinase enzyme represents an attractive and selective target in the field of the medicine, cosmetics and bio-insecticides. In this paper, experimental kinetics and computational analysis were used to study the inhibition of tyrosinase by analogs of Kojic acid. The main interactions occurring between inhibitors-tyrosinase complexes and the influence of divalent cation (Cu^2+^) in enzymatic inhibition were investigated by using molecular docking, molecular dynamic simulations and electrostatic binding free energy by using the Linear Interaction Energy (LIE) method. The results showed that the electrostatic binding free energy are correlated with values of constant inhibition (*r*^2^ = 0.97).Thus, the model obtained here could contribute to future studies of this important system and, therefore, eventually facilitate development of tyrosinase inhibitors.

## 1. Introduction

**Figure 1:**
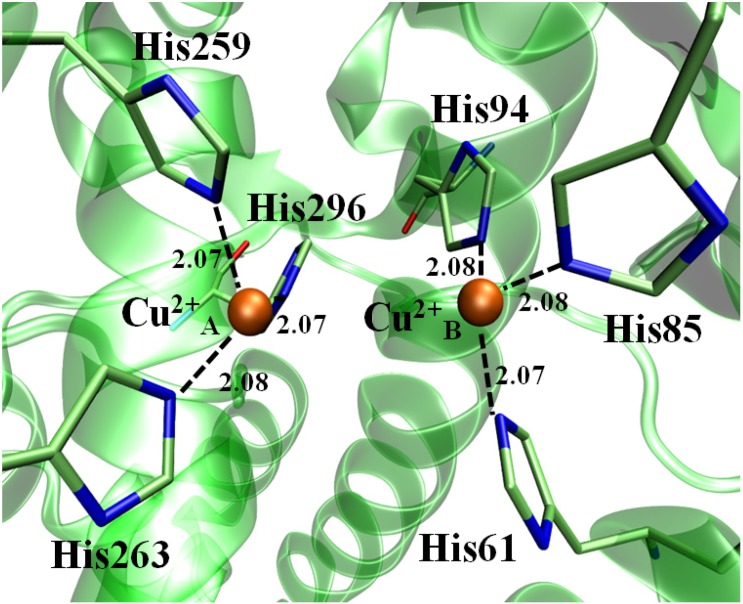
3D perspective of *Ab*TYR catalytic site containing two divalent copper cations chelated by His61, His85, His94, His259, His263 and His294 catalytic aminoacid residues. All distances are in Å.

According to the World Cancer Report, skin cancer constitutes 30% of all newly diagnosed cancers in the world [1]. Melanoma is one of the most serious consequences of skin cancer where melanocytes proliferate actively with enhanced accumulation of melanin pigment, leading to pigmentation and discoloration of the skin, in addition to tumor formation. Up-regulated levels of tyrosinase enzyme seem to contribute significantly to the enhanced synthesis and accumulation of melanin in melanocytes [2]. Tyrosinase has an important role in the pathway of melanin biosynthesis [3], melanin synthesis in skin [4,5] and wound healing [6,7]. Structurally, tyrosinase belongs to type-3-copper-protein family [8], with two copper ions (Cu^2+^), each coordinately bound to a distinct set of three histidine residues, spatially oriented within the active site due to the presence of two coupled copper cations, which are able of to activate dioxygen to initiate catalytic activity (Figure 1) [9].

Excessive accumulation of melanin, due to the overexpression of enzyme, leads to skin disorders such as age spots, freckles and malignant melanoma [10,11]. Tyrosinase may also be related to potential production of neurotoxicity by synthesizing dopamine-quinones, which contribute to neurodegeneration associated to Parkinson’s disease [12]. In addition to its catalytic features, tyrosinase is distinctive from other enzymes because it displays various inhibition patterns. Recently, the 3D structure of *A. bisporus* Tyrosinase (*Ab*TYR) in complex with the tropolone inhibitor was experimentally obtained and deposited in Protein Data Bank (PDB) with access code 2Y9X. Several kinetic and computational studies on tyrosinase inhibition mechanism have been published [13,14,15,16,17,18,19]. Besides, TYR inhibition has also been extensively studied for cosmetic, medicinal, and agricultural purposes [20,21].

**Table 1:** General informations about *Ab*TYR inhibitors analyzed in this study.

Inhibitor	2D Structure	*Ki* Value
INH1	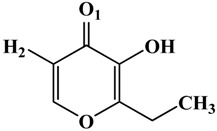	1 mM
INH2	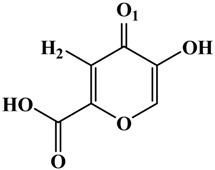	145 µM
INH3	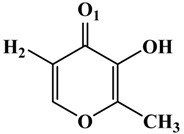	ND *
INH4	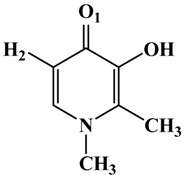	64 µM
KA	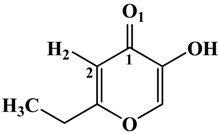	5 µM
Tropolone	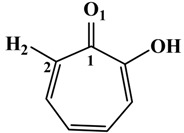	0.8 µM **

* Not Detected. ** Experimental value obtained by Espín and Wichers [22].

A number of tyrosinase inhibitors have been discovered and majority of them consist of the a phenol structure or of metal chelating agents [23,24]. These compounds represent one of the most promising classes of tyrosinase inhibitors in terms of potency, as with Kojico Acid (KA). KA is a metabolite produced mainly by fungi belonging to *aspergillus* genera. This natural product presents a wide range of pharmacological profile such as skin-whitening, antioxidant, anti-tirosinase and anti-tumor [23,24,25]. Recently, we have reported a novel function of KA as a macrophage activator [26]. In this paper, the interaction mechanism of inhibition was investigated with KA, tropolone and four KA analogs: 2-Ethyl-3-hydroxy-4H-pyran-4-one (INH1), 5-Hydroxy-4-oxo-4H-pyran-2-carboxylic acid (INH2), 3-Hydroxy-2-methyl-4-pyrone (INH3) e 3-Hydroxy-1,2-dimethyl-4(1H)-pyridone (INH4) through computational simulation and kinetics analysis (Table 1).

## 2. Results and Discussion

### 2.1. Experimental Kinetic Assays of Tyrosinase and Inhibition by KA Analogs

In order to show Michaelis constants *K*m, and *V*max, the kinetic analysis was determined from the equation of the Lineweaver-Burke plot. The inhibition constant (*Ki*), which is the concentration of inhibitor required to KA analogs (INH1, INH2, INH3 e INH4) to inhibit 50% of reaction.

**Figure 2:**
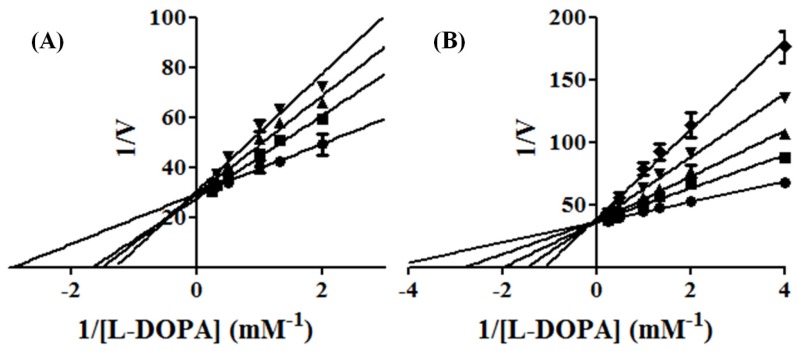
Lineweaver-Burk plot on the oxidation of L-DOPA by tyrosinase with INH2 (**A**: 0 [●]; 0.05 [■]; 0.1[▲] and 0.2 [▼] mM) and with INH4 (**B**: 0 [●]; 0.025 [■]; 0.5 [▲]; 0.1 [▼] and 0.2 [♦] mM).

The kinetic parameters showed that the maximum velocity *V*_max_ of the reaction was unchanged, while the apparent affinity *K*m was increased to INH2 e INH4 inhibitors (Figure 2). Kinetic studies pointed those inhibitors INH2 and INH4 showed competitive inhibition, since higher concentrations of L-DOPA reversed the inhibitory effect of the INH2 and INH4. The Ki’s of two compounds (INH4 and INH2**)** were 64 μM and 145 μM, respectively. These results match the values of affinity enzyme-inhibitor (*Ki*) and the values of free interaction energy. It is likely that these inhibitors present those kinetics parameters for showing similar features derived of inhibitors with chelanting and competitive properties [22].

**Figure 3:**
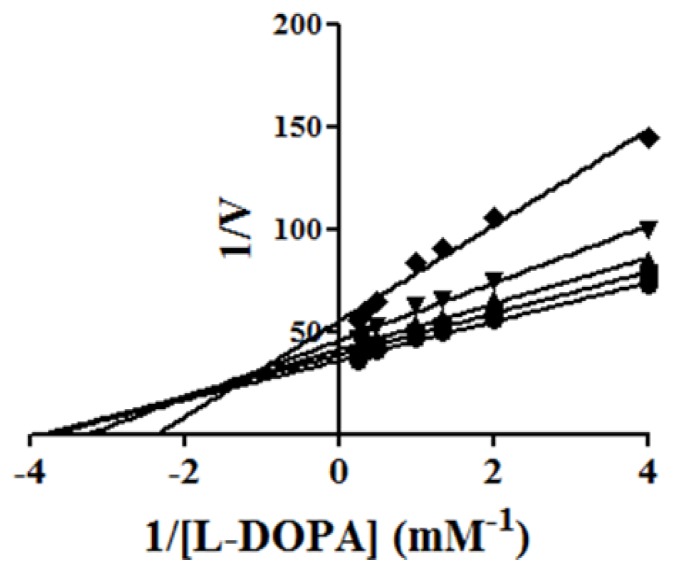
Lineweaver-Burk plot on the oxidation of L-DOPA by tyrosinase with INH1 (0 [●]; 0.2 [■]; 0.4 [▲]; 0.8 [▼] and 1.6 [♦] mM).

The inhibitor INH1 (Figure 3), showed a type of mix inhibition (competitive and non-competitive) with *Ki* = 1 mM, while the inhibitor INH3, it did not show any kinetic inhibition, suggesting that methyl group of this inhibitor shows less interactions with the amino acid near the enzyme site.

### 2.2. Molecular Docking

Previous molecular docking studies have been applied to elucidate the interactions occurring in Tyrosinase complex with isophthalic acid [21], hesperetin [9] and oxymatrine [27]. In order to perform similar analysis, KA analogs showed in experimental section were submitted to molecular docking calculations. In order to evaluate the MolDock Score implemented in Molegro Virtual Docking program (MVD), a re-docking procedure was carried out using the crystal inhibitor (Tropolone) coordinates as reference. Then, a comparison of orientation and conformation of tropolone inhibitor in the crystal structure of the *Ab*TYR, as proposed here, revealed an excellent agreement for the previous theoretical models obtained by molecular docking. In this way, the molecular docking was used to determine the position and conformation of KA and its analogues in the receptor-binding pocket of the *Ab*TYR.The re-docking results show that tropolone conformation obtained by docking is completely superimposed in tropolone experimental conformation. These results are shown in Supplementary Figure S1, where the tropolone conformation obtained by molecular docking is completely superimposed in tropolone experimental conformation. Besides, the KA analogs were also submitted at same docking procedure, the results show these compounds were docked in same region than tropolone inhibitor, in accordance with our experimental results, were the inhibitory action for these compounds are in competitive mode (see Supplementary Figure S2). All inhibitors conformations can be seen in support information. These results suggest that the MVD software reproduced the top conformation of experimental compounds inside the binding pocket of the *Ab*TYR catalytic site.

Previous computational studies showed that Met280 and Asn260 residues are involved in the first stage of inhibitors recognition and their fixation during docking and MD simulations [21,28], respectively. In these studies, the *Van der Waals* interactions are promoted by Met280 and Asn260 residues, these interactions occurring when these residues are contacting to compounds that are founded in tyrosinase catalytic site [3,9,20,21]. In our docking simulations, the same *Van der Waals* interactions could be founded, the CH_3_S group of Met280 interacts with carbonyl group in all KAD compounds and the carbamoyl group of Asn260 interacts with apolar groups in *para* position of all KA analogs.

About the interactions highlighted previously, we can highlight the one between Asn260 residue and the active compounds INH2 and INH4 with distances less than 2 Å. Table 2 shows the inhibitors distances between Asn260 and the cooper ions (Cu^2+^ A).On the other hand, the inhibitor INH1, which plays a role in preventing the entry of the substrate in the active site, exerting a mixed inhibitory action, presented distance greater than 2 Å concerning the Asn260.

**Table 2:** Atomic distances obtained by molecular docking procedure. The atoms O1 and H2 were numbered using 2D structure of KA as show in Table 1. All distances are calculated in Å.

KA Analogue	Atom	*Ab*TYR Atom	Distance
INH1	O1 H2	Cu^2+^ A OD1 (Asn260)	3.69 2.91
INH2			3.50 1.82
INH3			3.84 2.04
INH4			3.57 1.74

Table 3 shows the values of energy of affinity of the enzyme-inhibitor complex obtained using MVD software. The results demonstrate some variation of energy values; however, the inhibitors that have a higher number of hydroxyl groups achieved better affinity values with the enzyme. These energy scores suggest that the inhibitors INH2 and INH4 exhibit behavior similar to that of Kojic acid, emphasizing its inhibitory activity of a competitive nature, as well as their interaction with one of the copper ions (Cu^2+^ A). These data indicate that the region occupied by the inhibitors is similar to that found in previous studies [9,28], *i.e.*, inhibition by competition with the substrate L-DOPA in the cavity of the active site.

**Table 3:** Docking energy results obtained using MVD program and MolDock score function.

Inhibitor	MolDock Score (kcal·mol^−1^)	Type of Inhibition
KA	−11.81	Competitive
INH1	−12.05	Mix
INH2	−14.10	Competitive
INH3	−11.33	ND *
INH4	−13.08	Competitive

* Not Detected.

### 2.3. Molecular Dynamics (MD) and LIE

As explained in theoretical section, all inhibitors, including crystal inhibitor (Tropolone), were subjected to molecular dynamics simulations for a period of 2 ns. During the MD simulation, the inhibitors remained in the active site by performing interactions with copper ions and the amino acid residues constituting the active site. The proposed inhibitors keep similar interactions to those found in previous studies [10,21,28] with His61, His85, His94, His259, His263 and His294 residues which constitute the active site. The all histidine residues were also stable during MD, leaving in all the complexes studied an average distance of 2.075 Å ± 0.005 (Table 4) to the copper ions, confirming previous studies which indicate the maintenance of the initial coordinates during the study dynamics [21].

**Table 4:** Average distances between His residues and Cu^2+^ cations in their respectively coordination sphere during MD simulations. All distances are calculated in Å.

His Residue	Cu^2+^ ion	Atomic Distance
His60(NE2)	Cu^2+^ A	2.07
His84(NE2)		2.08
His93(NE2)		2.08
His258(NE2)	Cu^2+^ B	2.07
His262(NE2)		2.07
His295(NE2)		2.08

The ligands INH2 and INH4 interacts directly with the copper ion coordinated by His259, His264 and His296, causing a break in the link between the two copper ions, providing chelation, causing the copper ion which had an average distance of 2.07 Å change to a distance of 3.50 Å of His residues (259, 264 and 296). The inhibitor INH1 only positioned in the catalytic cavity of the active site, making interactions only with residues Asn260 and Met280, with no visible interaction with either copper ions. Previous studies recognize compound structures for hydroxyl groups as powerful inhibitors of tyrosinase [3,20,22,29,30]. These groups tend to perform nucleophilic attack to copper ions which make up the active site, destabilizing them through the exchange of protons during enzyme catalysis, resulting in inactivation of tyrosinase [31]. About, INH3 compound, it has show no strong influence in catalytic site, which can explain the reason it has the lowest activity in all KA analogs compounds. The root mean square deviation (RMSD) for all inhibitors are summarized in Supplementary Table S1.

The enzymatic complexes containing ligand-enzyme, as well as ligand in solution only, were subjected to the molecular dynamics calculations described above, allowing the free energy (FE) to be calculated. The complex tyrosinase-INH4 had a calculated energy by LIE method of −5.76 kcal·mol^−1^, getting a difference of 0.14 kcal·mol^−1^ from the experimentally calculated value which was −5.62 kcal·mol^−1^. Moreover, the affinity of the complex INH2-tyrosinase was experimentally calculated to −5.23 kcal·mol^−1^, getting a difference of 1.17 kcal·mol^−1^ from the value calculated by the LIE methodology (−6.40 kcal·mol^−1^).

The values of binding affinity, derived from molecular dynamics trajectories (MD) by LIE method, are shown in Table 5 and are in good agreement with the experimental data produced in this study. To give more reliable results and enhance the robustness of the methodology used for the prediction of the affinity of the ligands studied, we extended this methodology to two well-known inhibitors of this enzyme: kojic acid and tropolone, with errors calculated for both, ranging from −0.23 kcal·mol^−1^ to −0.16 kcal·mol^−1^, respectively, which is satisfactory, compared to previous works [32,33].

**Figure 4:**
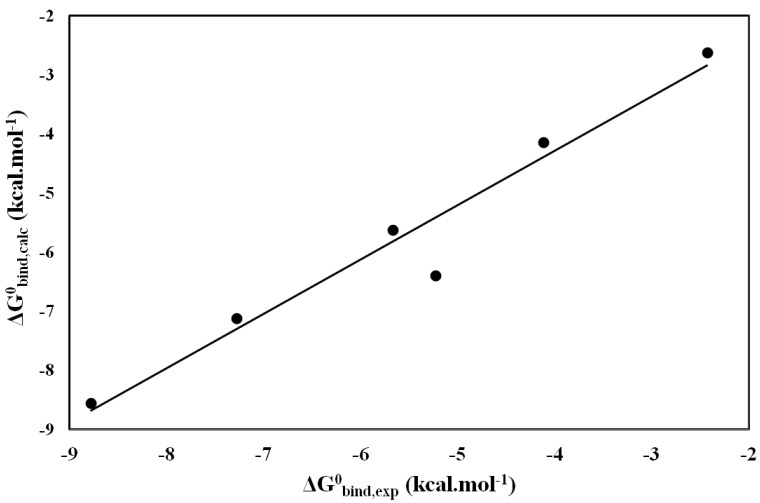
Linear correlation graphic between 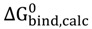 and 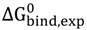 (energy values in kcal·mol^−1^).

The absolute binding calculated for the Tropolone and Kojic acid inhibitors using LIE (−8.55 and −7.12 kcal·mol^−1^, respectively) are in agreement with the experimental values (−8.78 and −7.28 kcal·mol^−1^, respectively). On the other hand, the absolute binding calculated for the INH2 inhibitor using the same methodology (−6.40 kcal·mol^−1^) reproduces the experimental affinities (ΔG°_bind,exp_ = −5.23 kcal·mol^−1^) with an error of 1.17 kcal·mol^−1^. In general, the calculations of bind affinities from MD trajectories through the LIE method are in excellent agreement with experimental data (Figure 4). The INH3 inhibitor showed the lowest affinity to *Ab*TYR enzyme (ΔG°_bind,exp_ = −2.62 kcal·mol^−1^), experimentally it was not possible to identify the reason of this lower affinity; however, our MD simulations revealed that this inhibitor tends to leave the enzyme catalytic site, what can justify its weak binding. From comparison between the theoretical predictions and the experimentally measured kinetic data for all molecules, it seems that the free energy calculations are reliable for practically all analogs of KA in complex with the *Ab*TYR enzyme. The coefficient of determination obtained for the linear fit of predicted values *versus* the observed contributions was good (*r*^2^ =0.97).These results suggest that the standard parameterization of LIE is a robust method that reproduces the experimental affinities.

**Table 5:** Free energy terms values for LIE calculated for each enzyme-inhibitor complex and its experimental energy values *.

Inhibitors	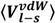 *_bind_*	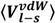 *_free_*	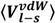 *_bind_*	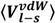 *_free_*	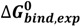	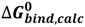
Tropolone	−17.89	−10.39	−55.12	−35.68	−8.78	−8.55
KA	−22.9	−9.57	−47.13	−32.86	−7.28	−7.12
INH1	−18.85	−12.77	−26.54	−18.32	−4.12	−4.14
INH2	−21.79	−9.71	−52.83	−40.08	−5.23	−6.40
INH3	−18.14	−11.08	−23.21	−19.59	−2.43	−2.62
INH4	−19.53	−11.84	−42.23	−30.79	−5.76	−5.62

* All equation terms and energy values in kcal·mol^−1^.

## 3. Experimental

### 3.1. Experimental Section

#### 3.1.1. Tyrosinase Activity

Kojic acid (KA) and its analogues (2-Ethyl-3-hydroxy-4H-pyran-4-one (INH1); 5-Hydroxy-4-oxo-4H-pyran-2-carboxylic acid (INH2); 3-Hydroxy-2-methyl-4-pyrone (INH3) and 3-Hydroxy-1,2-dimethyl-4(1H)-pyridone (INH4), L-DOPA and enzyme mushroom tyrosinase were obtained from Sigma-Aldrich. These reagents for anti-tyrosinase assay were of AR grade. Tyrosinase-inhibition activity of the analogues INH1, INH2, INH3 and INH4 were performed by using L-DOPA as a substrate according to Kubo *et al.* [34] with slight modification. Incubation was carried out at 160 µL of different concentrations of the substrate L-DOPA, 20 µL (2.4 U) of enzyme mushroom tyrosinase and 20 µL of different concentrations of KA and its analogues.

All solutions were prepared in Phosphate Buffered Saline (PBS) pH 7.2. The reaction was initiated by addition of enzyme to all wells simultaneously. The change in absorbance due to the formation of dopachrome (final product) was assessed during the first 5 min in the microplate reader with 490 nm filter.

#### 3.1.2. Determination of Inhibition Constant (*Ki*)

The mode of inhibition on the enzyme was assayed by Lineweaver-Burk plot. The assay varied the concentracion of L-DOPA (0.00, 0.25, 0.50, 0.75, 1.00, 2.00, 3.00 and 4.00 mM) and inhibitor (0.00, 0.025, 0.05, 0.10, 0.20, 0.40, 0.80, 1.60 and 3.00, 2.00 mM). The kinetic parameter *Ki* was determined with the GraphPad Prism^®^ 5.0 software, according to the equations follows:

- Competitive Inhibition:

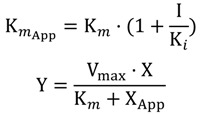
- Mixed Inhibition:

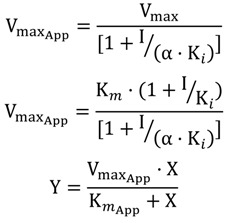

where Y, X and I denotes average absorbance change *per* minute, concentration of l–DOPA and concentration of KA analog, respectively. The parameter α determines mechanism, its value determines the degree to which the binding of inhibitor changes the affinity of the enzyme for substrate.

### 3.2. Computational Section

#### 3.2.1. Molecular Docking

All molecular docking calculations were performed using as staring point the 3D structure of *A. bisporus* Tyrosinase (*Ab*TYR) was obtained from the Protein Data Bank (access code 2Y9X), a repository for structural data of large biological molecules, such as proteins and nucleic acids [19]. The crystal inhibitor (Tropolone) and KA and its analogs were submitted to calculations docking studies using Molegro Virtual Docker (MVD) software [35,36]. The following parameters were used for the guided differential evolution algorithm: population size = 100, crossover rate = 0.9, scaling factor = 0.5, and max evaluations = 2500. The algorithm used in the docking studies was the MolDock Score, which is an adaptation of the Differential Evolution (DE) algorithm. The MolDock Score function was used because it yields a higher docking accuracy than other state-of-the-art docking algorithms (MVD: 87%, Glide: 82%, Surflex: 75%, FlexX: 58%) [36].

The MVD program [36] used to perform the docking study apply the MolDock algorithm, which is based on a new hybrid search algorithm, called guided differential evolution. The guided differential evolution algorithm combines the differential evolution optimization technique with a cavity prediction algorithm [36]. The docking scoring function of MolDock is based on a piecewise linear potential (PLP), introduced by Gehlhaar *et al.* [37,38] and further extended in GEMDOCK by Yang *et al.* [39].

The MolDock Score function (E*_score_*) is defined by the following energy terms:

E*_score_* = E*_inter_* + E*_intra_*

where E*_inter_* is the ligand-protein interaction energy and E*_intra_* are the internal energy of the ligand. The E*_inter_* is determined by follow equation:

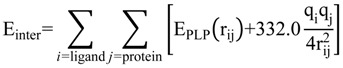



The E_PLP_ term is a piecewise linear potential using two different sets of parameters: one set for approximating the steric (van der Waals) term between atoms and the other stronger potential for hydrogen bonds [36]. The E*_intra_* is calculated by the following equation:

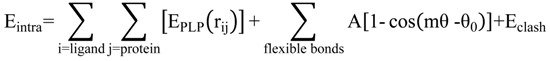



The double summation calculates all the energy terms involving pairs of atoms of the ligand, except those connected by two bonds. The second summation calculates the torsional energy, where θ is the torsional angles of the bond. The average of the torsional energy term contributions is used if several torsions can be determined. The last term, *E*_clash_ assigns a penalty of 1000 if the distance between two atoms (more than two bonds apart) is less than 2.0 Å. Thus, the *E*_clash_ term punishes infeasible ligand conformations.

#### 3.2.2. Molecular Dynamics Simulations and Free Energy Binding

The empirical scoring function used by MVD program is fast to be performed; it treats contributions to the binding free energy, e.g., entropy and solvation, in a very approximate fashion, which makes it difficult to obtain accurate predictions. Then, we decided to perform molecular dynamics simulations in order to accurately describe receptor flexibility and salvation around each inhibitor and to determine their binding free energy.

Molecular dynamics simulations and LIE calculations were performed with the package Q [40]. The MD simulations were conducted in a sphere of radius 25 Å, centered on the ligand. Since the standard pKa values of ionizable groups can be shifted by local protein environments [41], the protonation state of all residues was calculated at pH = 7 using the empirical PropKa server [42]. Ionizable residues close to the ligand were charged, and residues close to the boundary edge were made neutral. The sphere was solvated with TIP3P water molecules [43] and slowly heated to 300 K. The system was equilibrated for 50 ps at 300 K to allow full relaxation of the system before snapshot collection. The collection phase was 2000 ps with a 1 fs time step, and snapshots of the configuration were saved every 25 fs. Water molecules at the sphere boundary were restrained in order to mimic the dipole distribution of bulk water through the SCAAS model [44] and the SHAKE algorithm was used to constrain the geometry of the water molecules and solute bonds involving hydrogens [45]. The nonbonded cutoff was set to 10 Å for all atoms, with the exception of ligand atoms, for which no cutoff was applied. Besides the cutoff, long-range electrostatic interactions were treated using the Local Reaction Field (LRF) multipole expansion approximation [46]. Simulations of the ligands in the free state were performed under similar conditions in a sphere of water. All classical molecular parameters were assigned according to the OPLS-AA force field [47]. Besides, in order to maintain the coordination sphere in Cu^2+^ ions for enzyme system, a constrain was applied using a force constant of 600.0 kcal·mol^−1^.Å^2^ during all MD simulation steps.

The LIE method was used to calculate binding free energies from the MD simulations [32,48]. The method is a semi-empirical scoring function based on linear free energy relations. Ligand surrounding energies (*l* − *s*) were extracted from the collection phase MD trajectories, and binding free energies were calculated according to:

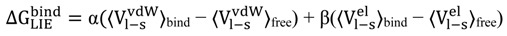

where ⟨⟩ denotes averages of the van der Waals (*vdW*) and electrostatic (*el*) interaction energies. The parameter α has been empirically determined to 0.181 [40] and β is a theoretically derived parameter which varies depending on the chemical nature of the ligand; for neutral ligands containing one hydroxyl group, it was used 0.37 and for neutral ligands containing two hydroxyl group, it was used 0.33 [40]. The same set of parameters was used to calculate binding free estimates for the correct and wrong conformations.

Finally, binding free energy estimates were compared to experimentally measured *Ki* values, which were converted into experimental free energy values using the equation:

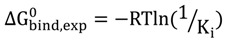



## 4. Conclusions

In this report, experimental kinetic assays were coupled with computational study, applying molecular docking, molecular dynamics simulations and binding free energy to provide insights into the activity of inhibitor against tyrosinase enzyme. Kinetic studies showed that inhibitors INH2 and INH4 are competitive, while the inhibitor INH1 showed a mixed-type of inhibition. In addition, the inhibitor INH3 did not show any kinetic inhibition. The computational simulation supports these findings. The analysis of individual interactions between the inhibitor and the amino acids of the enzyme active site reveals how the influence of Met280, Asn260, His61, His85, His94, His259, His263 and His294 residues seems to be crucial, being especially important the interactions established between the inhibitor and theses residues. Finally, comparing the theoretical predictions on complexes with the experimentally measured kinetic data, *Ki*, it seems that the ligand affinity obtained through LIE calculations is in excellent agreement with experimental data. Thus, a combination of inhibition kinetics and computational modeling may facilitate the testing of potential tyrosinase inhibitors and the prediction of their inhibitory mechanisms.

## Supplementary Materials

Supplementary materials can be accessed at: http://www.mdpi.com/1420-3049/19/7/9591/s1.
