# Supplementary material for: Combined Kinetic Studies and Computational Analysis on Kojic Acid Analogs as Tyrosinase Inhibitors
Source: Molecules. 2014 Jul 7;19(7):9591–605. doi: 10.3390/molecules19079591 (PMC6271673; doi:10.3390/molecules19079591)

Supporting Information

Table S1. Root mean square deviation (RMSD) obtained by MD simulations for all inhibitors. The standard deviations are in parenthesis.

Inhibitor	RMSD (Å)
Tropolone	1.02 (0.18)
KA	0.67 (0.27)
INH1	0.91 (0.23)
INH2	1.28 (0.13)
INH3	1.87 (0.36)
INH4	0.74 (0.37)

Figure S1. Re-docking result of crystal inhibitor obtained by MVD software.

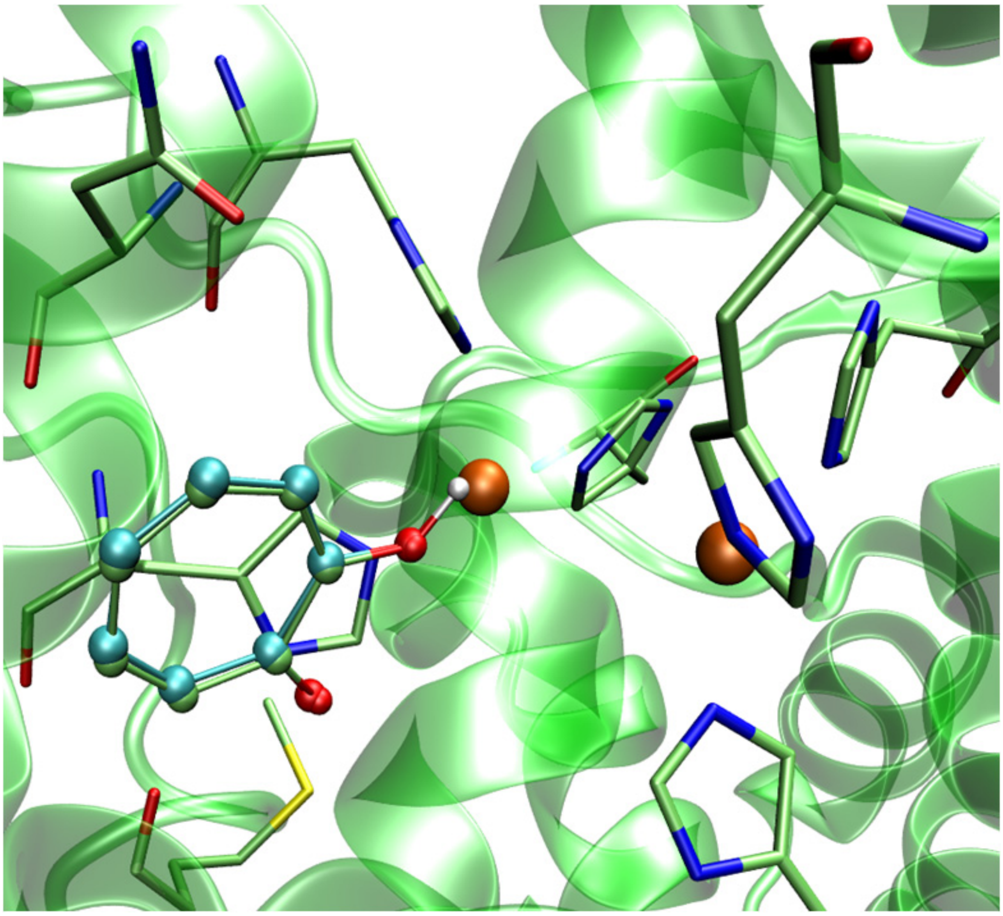

Figure S2. Docking results of Kojic Acid and its analogous obtained by MVD software.

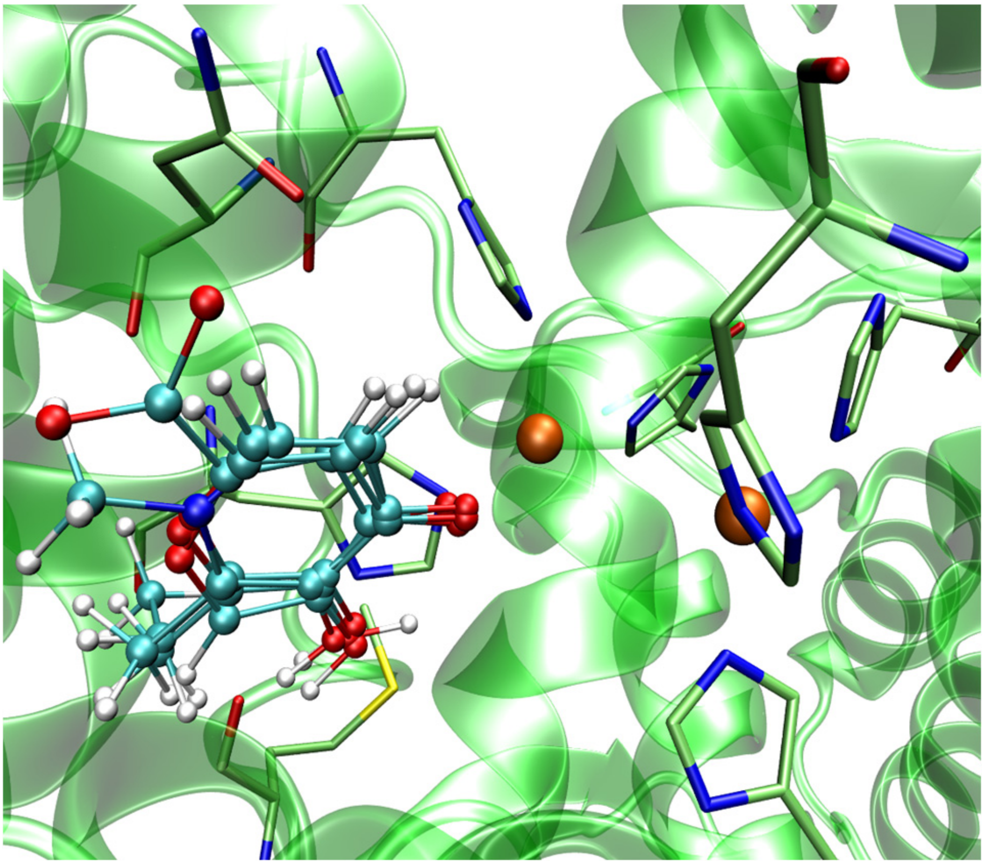

Supplement: Supplementary file 1 [file molecules-19-09591-s001.pdf]
